# Bacterial Colonization on Healthcare Workers’ Mobile Phones and Hands in Municipal Hospitals of Chongqing, China: Cross-contamination and Associated Factors

**DOI:** 10.1007/s44197-022-00057-1

**Published:** 2022-09-07

**Authors:** Ning Yao, Xue-Fan Yang, Bing Zhu, Chun-Yan Liao, Ya-Ming He, Jiang Du, Nan Liu, Chun-Bei Zhou

**Affiliations:** 1 Division of Expanded Program On Immunization (EPI), Chongqing Center for Disease Control and Prevention, Chongqing, China; 2 Department of Disinfection and Vector Control, Chongqing Center for Disease Control and Prevention, Chongqing, China; 3 Department of Microbiology, Chongqing Center for Disease Control and Prevention, Chongqing, China

**Keywords:** Bacterial contamination, Healthcare workers, Hospital-acquired infection, Mobile phone

## Abstract

**Background:**

Mobile phones are widely used in clinical settings and could be colonized by potential pathogenic bacteria which may lead to hospital-acquired infections (HAIs) transmission. This study aimed to determine the prevalence of bacterial contamination of healthcare workers’ (HCWs) mobile phones, identify bacterial isolates, and assess the factors associated with mobile phone contamination.

**Methods:**

Self-administered questionnaire was used to collect the information on the demographic characteristics and the use of mobile phones. A total of 111 HCWs’ hands and their mobile phones were swabbed, then bacterial culture, isolation, and identification were performed. Univariate and multivariable logistic regression were applied to identify factors associated with mobile phone bacterial contamination.

**Results:**

Totally 106 (95.5%) of the 111 mobile phones investigated were contaminated with bacteria. *Staphylococcus epidermidis* (13/111), *Acinetobacter baumannii* (4/111) and *Staphylococcus aureus* (3/111) were the predominant bacterial isolates from HCWs’ mobile phones. Univariate analyses showed that age, gender, profession and the frequency of mobile phone utilization were significantly associated with the number of bacterial colonization. Frequency of phone utilization (OR 8.366; 95% CI 1.496–46.797) was found to be the most significant factors associated with the qualified rate of mobile phones bacterial load. In addition, phone cover using was associated with the increased risk of mobile phone bacterial contamination.

**Conclusion:**

There was cross-contamination between hands and phones. It is necessary to develop guidelines for mobile phone cleaning. Special attention needs to be paid to the disinfection of mobile phone covers to reduce contamination and transmission of pathogens.

**Supplementary Information:**

The online version contains supplementary material available at 10.1007/s44197-022-00057-1.

## Introduction

Hospital-acquired infection (HAI) is the most frequent result of unsafe patient care worldwide [1], and it is deemed a global public health problem [2–4]. Not only patients, but also health care workers (HCWs) can have the possibility of acquiring and transmitting the infection in hospitals [5, 6]. HAIs give rise to increasing morbidity, mortality, length of stay, and hospital costs in many countries including China [7]. It is critical to control HAIs as the world battles with the COVID-19 pandemic.

Vast majority of HAIs are caused by bacteria, and it is well recognized that certain environments may facilitate the transmission of healthcare-associated pathogens [8]. HCWs’ fomites are highly predisposed to bacterial contamination in health care settings and are potential sources of HAIs [9]. Contaminated hands of HCWs play a critical role in spreading HAIs in hospitals [10]. Objects with frequent hand contact, such as stethoscopes and mobile phones, could serve as reservoirs from which HAIs can spread to the hands of HCWs and then to patients [11]. In health services, mobile phones are not only used in traditional fields such as registration and medical treatments but also used in healthcare facilities for better disease control [12]. Existing data have demonstrated the bacterial contamination of mobile phones in clinical settings and reported that the levels of contamination and the types of bacteria depended on the settings [13]. Some microbiologists share the opinion that the combination of constant handing and heat generated by mobile phones has created prime breeding ground for all sorts of microorganisms [12]. Previous studies described that microorganisms isolated from mobile phones and HCWs’ hands were similar [14, 15], and the mobile phone bacterial contamination rate ranged from 40 to 100% [16]. It was estimated that averagely a mobile phone harbored 25,107 bacteria per square inch [17], and mobile phones could be also colonized by fungi and even RNA viruses, such as SARS-CoV-2 [18]. A review of surveys showed that the rate of bacteria isolated from the mobile phones in descending order were *Coagulase-negative staphylococci* (*CoNS*), *Micrococcus*, *Viridans streptococci,* and *Acinetobacter species* [11]. The risk of cross-infection cannot be ignored if the HCWs are still using contaminated mobile phones without hand hygiene before medical activities.

Our study aimed to assess the prevalence of bacterial contamination on both HCWs’ mobile phones and their hands, identify the bacterial species, and define the factors associated with bacterial isolates from the mobile phones of HCWs working at municipal hospitals of Chongqing, China. The results may provide more evidence to support the promotion of HAI control.

## Methods

### Study Participants and Sampling Methods

This cross-sectional study was conducted from April to June in 24 hospitals of Chongqing, China. Sample size was calculated based on a previous similar study [19]. We used the formula *N*=${\mathrm{Z}}_{\mathrm{\alpha }/2}^{2}$ *P*(1−*P*)/*d*^2^, where *N* = sample size, *Z*_α/2_ = statistic for the level of confidence = 1.96,*α* = 0.05, *P* = expected prevalence = 50% (prevalence of mobile phone contamination with bacteria) [16], and *d* = allowable error = 0.1(10%). The calculated sample size was 96. Considering a 25% (24) non-response rate, the total sample size was 120. The study population was health care professionals working in nine different departments with high prevalence of HAIs, including nurses, doctors, laboratory technicians, healthcare assistants, and other HCWs. According to the degree of cleanliness requirement, these departments were divided into 4 types of environments: Class I (cleanliness best) included laminar flow operating room. Class II (cleanliness better) included general operating room, ICU, delivery room, and neonatal ward. Class III (cleanliness good) included hemodialysis room and central sterile supply department. Class IV (cleanliness normal) included consulting room for stomatology and therapeutic room. The sample size was allocated equally to the four environments to their total number. Only the mobile phones of the HCWs used for more than 1 week with the surface not being disinfected on the day of the investigation were selected as samples. A total of 120 HCWs were approached and invited to participate, and 111 of them agreed and completed the investigation (response rate = 92.5%).

### Data Collection and Laboratory Methods

A self-administered questionnaire was administered to all participants to collect the information on demographic characteristics (age, gender, profession, educational status and department), with questions regarding their use of mobile phones and awareness of the presence of microorganisms on mobile phones. All questions were formulated by referencing previously validated questionnaires [11, 20, 21]. Before the formal investigation, 20 pre-survey questionnaires were collected and reviewed in one of the 24 hospitals, and the questionnaire was adjusted and revised based on the pre-survey results. In formal investigation stage, further explanations were provided if the participants were confused or had any questions. After completing the questionnaire and sampling, a fixed researcher was asked to observe and record whether the HCWs used/touched their mobile phones within 10 min after hand hygiene. The observation was stopped after 10 min.

Hand samples were collected before hand hygiene. The participants were asked to have their five fingers closed together and a sterile swab moistened with sterile saline was used to rub back and forth twice from the finger root to the finger end (the area was approximately 30 cm^2^). Mobile phone samples were collected simultaneously with hand samples. The screens, earpieces, sides, and backs of mobile phones were swabbed using sterile swabs (the area was approximately 100 cm^2^). Strict bacteriological sample collection procedure was followed at the time of swabbing. Sample collectors wore masks and used quick-drying hand disinfectant to avoid cross-contamination. Each sample was given unique identification number and labeled with the name of the department. The sampling time was between 9:00 and 10:00 AM. The collected samples were kept in the brain heart infusion broth as transport medium and transported to the laboratory for culture within 5 h after sampling. Samples were mixed and immediately inoculated onto agar plates, which were incubated for 48 h at 36 ± 1 ℃ aerobically. Colony count was calculated using the semi-quantitative colony-forming unit (CFU) count method in which the number of colonies isolated from each sample was divided by the area sampled [20]. To obtain a higher purity of pathogenic bacteria, the methods of enriching, separating, and identifying were adopted. Samples were inoculated on blood agar, MacConkey agar, Salmonella-Shigella agar, and Mannitol salt agar aerobically at 37 ℃ for 18–24 h. Primary isolation of bacteria was made based on their colony characteristics and Gram stain reaction microscopically. Further identification was performed by conventional biochemical tests (like catalase test, oxidase test, coagulase test, carbohydrate fermentation, H_2_S production, citrate utilization test, motility, indole test, lysine decarboxylase test, lysine deaminase test, and urea test) and automated system VITEK 2 (BioMerieux, France). All methods were performed following relevant guidelines and regulations. Any sample with microbial growth was defined as contaminated. To evaluate the cleanliness of samples, we defined the qualified sample as bacterial colonies on hands/phones ≤ 10 CFU/cm^2^ according to the “Disinfection in Hospital Health Standards” (GB15982-2012) available in China. The qualified sample rate was defined as the sum of the qualified samples/total number of samples × 100%.

### Data Analysis

Median and interquartile range (IQR) was adopted for the statistical description of non-normal data. Differences in proportions between groups were assessed by Chi-Square test. Differences in the number of colonies among groups were assessed by non-parametric test including Wilcoxon test and Kruskal–Wallis test. The variables with statistical significance in univariate tests were further subjected to multivariable logistic regression analysis. The qualified rate of mobile phone of bacteria was the dependent variable. Differences were considered statistically significant at *p* < 0.05. All analyses were carried out using R (version 3.1.2, R Foundation for Statistical Computing, Vienna, Austria).

### Ethics Approval

The study was approved by the Ethics Committee of Chongqing Center for Disease Control and Prevention, and complied with Declaration of Helsinki. Participation in the survey was voluntary and anonymous. All participants who agreed to participate in this study gave their oral consents, but were blinded to the follow-up observations.

## Results

A total of 111 HCWs and 111 mobile phones belonging to them were assessed. The average age of the HCWs was 32 ± 9.0 years, mainly females (97; 87.4%) and nurses (85; 76.6%). The use rate of mobile phone was very high in hospital settings, high use (Once every 5 min) represented 10.8% of all participants, and moderate use (Once every 5–30 min) represented 21.6%. Seventy-eight of participants (70.3%) reported using mobile phone covers and 27 (24.3%) reported using phones at work, while 84 (75.7%) said that they never did so. However, of these 84 HCWs, 10 were found using/touching mobile phones in healthcare settings within 10 min after hand hygiene. Others were lost to follow-up or did not use/touch their phones. Seventy-one (64.0%) reported that it was forbidden to use mobile phones in their departments during work. Intriguingly, only two of them (2.8%) were observed to strictly obey the rules and put their phones at designated locations during the survey. Almost all participants (95.5%) believed that mobile phone should be cleaned and disinfected regularly, and wiping with alcohol was the most common methods of disinfection. The characteristics on the use of HCWs’ mobile phones and awareness of the presence of microorganisms on their phones are shown in Table 1.

**Table 1:** Characteristics on the use of HCWs’ mobile phones and awareness of the presence of microorganisms on mobile phones

Questions	Answers	*n* (%)
A smart phone or not	Yes	110 (99.1)
	No	1 (0.9)
Size of the phone screen	< 5.0 inches	68 (61.3)
	≥ 5.0 inches	43 (38.7)
Daily mobile phone usage time	< 1 h	12 (10.8)
	1–2 h	26 (23.4)
	> 2 h	73 (65.8)
Frequency of utilization	Once every 5 min	12 (10.8)
	Once every 5–30 min	24 (21.6)
	Once every 30–60 min	30 (27.0)
	Usually not, only when having calls	45 (40.5)
Application classification^a^	Making calls	103 (92.8)
	WeChat^b^	100 (90.1)
	Sending message	62 (55.9)
	QQ^c^	53 (47.8)
	Shopping	47 (42.3)
	Games	28 (25.2)
	Weibo^d^	27 (24.3)
	Else	1 (0.9)
Use of mobile phone at eating	Yes	40 (36.0)
	No	71 (64.0)
Use of mobile phone before sleep	Yes	97 (87.4)
	No	14 (12.6)
Use of mobile phone in the bathroom	Yes	51 (46.0)
	No	60 (54.0)
Use of a phone cover	Yes	78 (70.3)
	No	33 (29.7)
Depositary of mobile phone at work^a^	Pocket/trouser pocket	90 (81.1)
	Bag	53 (47.8)
	Desktop	32 (28.8)
	Drawer	6 (5.4)
Use of mobile phone at work	Yes	27 (24.3)
	No	84 (75.7)
Mobile phone use is banned in the department	Yes	71 (64.0)
	No	40 (36.0)
Frequency of mobile phone cleaning and disinfection	Regularly	36 (32.4)
	Occasionally	68 (61.3)
	Never	7 (6.3)
The way used in mobile phone disinfection^e^	Wipe with alcohol	78 (75.0)
	Wipe with wet tissue	21 (20.2)
	Wipe with a napkin	4 (3.9)
	Else	1 (1.0)
Hands cleaning while cleaning mobile phone^e^	Yes	81 (77.9)
	No	23 (22.1)
Do you think your phone is contaminated with bacteria?	Yes	109 (98.2)
	No	2 (1.8)
Do you think your phone should be cleaned and disinfected regularly?	Yes	106 (95.5)
	No	5 (4.5)
Do you think the phone should be banned at work in your department?	Yes	59 (53.2)
	No	52 (46.8)
Do you think the phone could cause HAI?	Yes	98 (88.3)
	No	13 (11.7)
Do you think the phone could spread diseases?	Yes	96 (86.5)
	No	15 (13.5)

^a^Multiple answers could be chosen

^b^WeChat is a multi-purpose app combining messaging, social media, and mobile payment in China

^c^QQ is an internet-based instant messaging software with functions such as online chat, video call, and file transfer

^d^Weibo is a social media platform based on user relations developed by the Chinese company Sina. It uses text, images, videos, and other multimedia forms to realize instant sharing and communication of information

^e^The total number of HCWs answered for this question was 104 (There were 7 people who had never cleaned or disinfected their phones)

The median of colony number of mobile phones was 2.9 (0.7–6.7) CFU/cm^2^. Out of these 111 mobile phones, 106 (95.5%) were contaminated, and the qualified rate of HCWs’ mobile phones of bacteria was 80.2%. The prevalence of mobile phone bacterial contamination was higher than that of hands, while the bacterial load on mobile phones was lower than that on hands (Table 2).

**Table 2:** Bacterial contamination of mobile phones and HCWs’ hands

Parameter	Mobile phones	Hands	*χ*^*2*^	*p*
Qualified rate (*n*/%)	89 (80.2)	47 (42.3)	33.48	< 0.01
Contamination (*n*/%)	106 (95.5)	96 (86.5)	5.50	0.02
Number of colonies: median (IQR) (CFU/cm^2^)	2.9 (0.7–6.7)	15.8 (0.8–60.8)	4.74^a^	< 0.01

*IQR* interquartile range, *CFU* colony-forming unit

^a^*Z* value

**Fig. 1:**
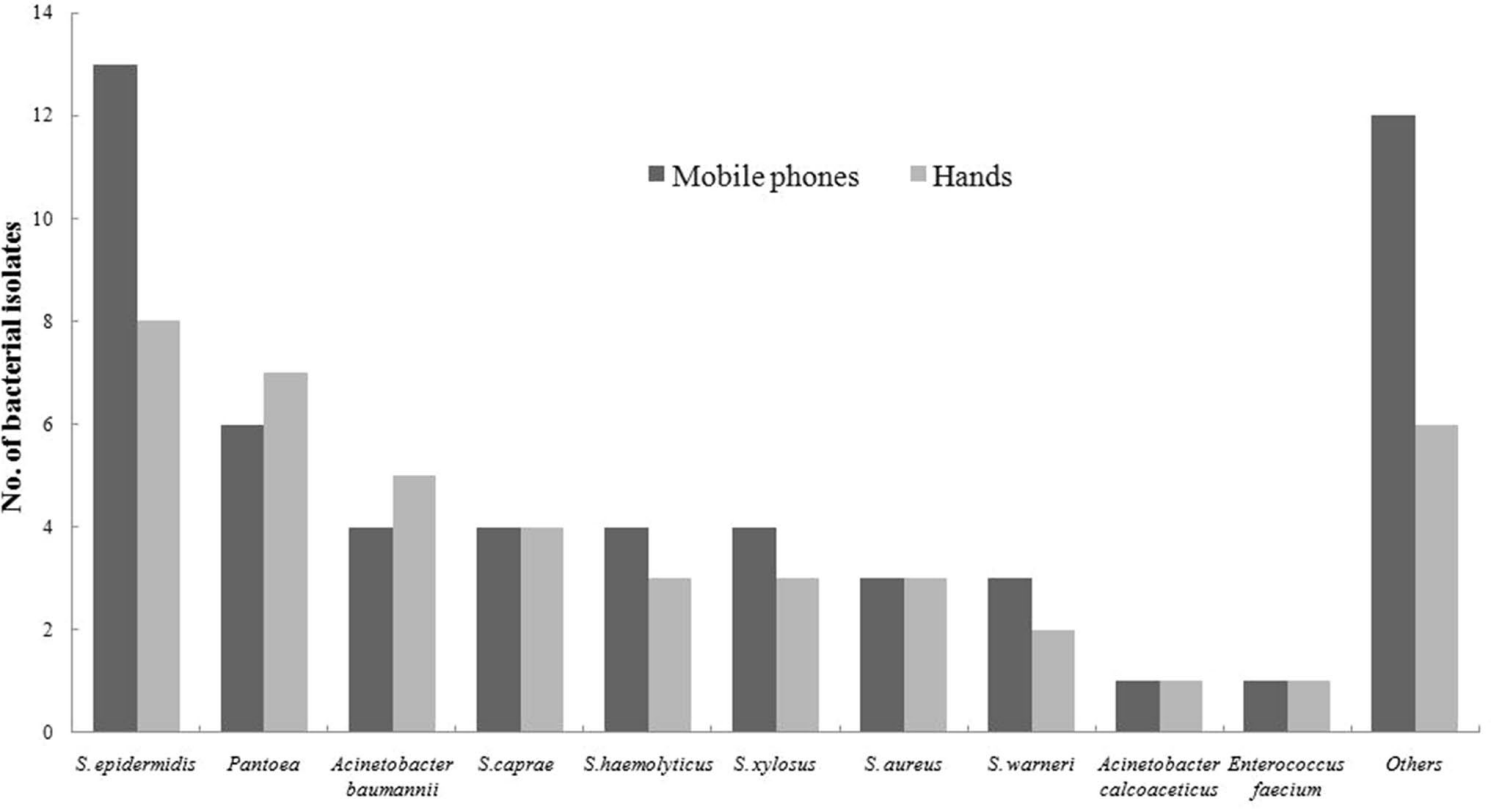
Organisms identified from HCWs’ mobile phones and their hands

Twenty-three kinds of bacteria were isolated from 54 HCWs’ hands or their phones, while no significant bacterial pathogens were isolated from the samples of the remaining 57 HCWs. The bacterial isolates from HCWs’ mobile phones were similar to those from their hands (Fig. 1). Twenty-one of 54 participants (38.9%) had at least 1 identical bacterium (including pathogenic bacteria and conditionally pathogenic bacteria) simultaneously isolated from their hands and mobile phones. It was found that 55 bacterial strains were isolated from 44 mobile phones and 43 bacterial strains were isolated from 36 hands. About 31.5% (35/111) of phones grew one bacterial species, 7.2% grew 2 different species and 0.9% grew 4 different species. *Staphylococcus epidermidis* (*S. epidermidis*) was the most common bacterium isolated from mobile phones, with 13 (11.7%) mobile phones contaminated. Other frequently isolated bacteria included *Pantoea* (6; 5.4%), *Acinetobacter baumannii* (4; 3.6%), *S. haemolyticus* (4; 3.6%) and *S. aureus* (3; 2.7%). In addition, *Escherichia coli* (*E. coli*) was isolated from a HCW’s mobile phone.

Results of the Chi-square test showed that the qualified rate of mobile phones bacterial load was significantly different between male and female HCWs (57.1% vs. 83.5%; *p* = 0.021), and the mobile phone qualified rate of nurses was higher than that of others (84.7 vs. 65.4%; *p* = 0.031). Those who used mobile phones more frequently had a lower qualified rate of mobile phone bacterial load. Non-parametric tests showed that the number of bacterial colonization varied between different ages, genders, and profession groups (*p* = 0.048, *p* = 0.004, and *p* = 0.045). The lower the frequency of mobile phone use, the fewer bacteria colonized on the surface (*p* = 0.005). The mobile phones with covers grew more bacteria than those without (*p* = 0.011) (Table 3). Different environments, educational levels, other mobile phone usage habits, and other variables were not significantly associated with the phone qualified rate of bacteria and the bacterial load (Table S1). Multivariable logistic regression analysis showed that, except the frequency of phone utilization (OR 8.366; 95% CI 1.496–46.797), all other included factors were not statistically associated with the qualified rate of mobile phone bacterial load (Table 4).

**Table 3:** Comparison of qualified rate and bacterial load of mobile phones according to demographic characteristics and usages

Characteristics and usage	No. of HCWs (*N* = 111)	No. of qualified	Qualified rate (%)	*χ*^*2*^	*p*	Median No. of colonies (IQR) (CFU/cm^2^)	*Z*	*p*
Age								
< 35 years	67	51	76.1	1.75	0.185	3.4 (0.8–8.4)	− 2.00	0.048
≥ 35 years	44	38	86.4			1.4 (0.5–4.7)		
Gender								
Male	14	8	57.1	5.35	0.021	8.9 (3.5–20.9)	2.94	0.004
Female	97	81	83.5			1.9 (0.7–4.9)		
Environment								
I	26	19	73.1	2.06	0.560	2.0 (0.4–5.5)	2.64^a^	0.450
II	32	25	78.1			1.7 (0.8–4.1)		
III	27	22	81.5			3.5 (1.1–8.2)		
IV	26	23	88.5			3.4 (0.6–5.9)		
Profession								
Nurse	85	72	84.7	4.68	0.031	2.1 (0.7–4.9)	2.03	0.045
Non nurse	26	17	65.4			4.2 (0.8–15.6)		
Educational level								
Bachelor’s degree below	43	33	76.7	0.52	0.470	2.9 (0.7–7.2)	0.44	0.662
Bachelor's degree or above	68	56	82.4			2.7 (0.7–6.6)		
Frequency of utilization								
Once every 5 min	12	7	58.3	9.09	0.028	4.7 (0.5–18.1)	12.79^a^	0.005
Once every 5–30 min	24	20	83.3			3.4 (0.5–6.4)		
Once every 30–60 min	30	21	70.0			4.6 (1.4–13.4)		
Usually not, only when having calls	45	41	91.1			1.4 (0.6–2.9)		
Use of a phone cover								
Yes	78	59	75.6	3.40	0.065	3.2 (1.2–8.2)	− 2.59	0.011
No	33	30	90.9			0.8 (0.4–3.8)		

IQR interquartile range; CFU colony-forming unit

^a^ The *χ*^*2*^ of Kruskal–Wallis test

**Table 4:** Factors associated with the qualified rate of mobile phones of bacteria among HCWs

Variable		*β*	*S*_*‾x*_	Wald	*p*	OR(95% Cl)
Intercept		− 1.500	0.892	2.810	0.094	
Age	≥ 35 years	0.911	0.580	2.473	0.116	2.488 (0.799–7.745)
Gender	Female	0.680	0.775	0.770	0.380	1.974 (0.432–9.021)
Profession	Nurse	0.763	0.663	1.325	0.250	2.145 (0.585–7.864)
Frequency of utilization	Once every 5–60 min	0.838	0.735	1.300	0.254	2.312 (0.548–9.760)
	Usually not, only when having calls	2.124	0.878	5.848	0.016	8.366 (1.496–46.797)
Use of a phone cover	No	0.933	0.625	2.227	0.136	2.543 (0.746–8.664)

## Discussion

Our investigation provided compelling evidence that HCWs’ mobile phones could be a potential reservoir for bacteria, including some pathogenic bacterial strains, as recognized by other scholars [22]. More interestingly, cover using was associated with  increased risk of mobile phone bacterial contamination.

The present survey showed that touch-screen smartphones were widely used by HCWs in municipal hospitals in Chongqing, and the frequency of usage was high and the cumulative time was long. The use of mobile phones was not only used to phone calls, but also with social contacts, payments, and entertainments as well as shopping*.* During the investigation, we have observed that individual HCWs of the key departments still used or touched mobile phones after hand hygiene. Therefore, it is particularly important to take hand hygiene again before patient contact. Although 64.0% of people confirmed that it was forbidden to use mobile phones at their departments during work, only 2.8% of them were observed to obey the rules, suggesting that the mobile phone ban at work was not strictly implemented. About 32.4% of the HCWs had the habit of cleaning and disinfecting mobile phones regularly, which was higher than those in similar researches of Morvai et al. [16] and Mark et al. [23], but lower than the result of Kotris I et al.[24]. This may be related to the constant emphasis on the importance of HAI control in recent years in China. Similar to other reports [19, 24], most HCWs wiped their mobile phone surfaces with alcohol. On one hand, alcohol is simple, cheap, easy to get, and will not damage their phones. On the other hand, a previous study has shown that regular decontamination of phones with alcohol can reduce contamination rates [22]. The HCWs were highly acquainted that pathogenic bacteria are colonized on mobile phone surfaces and may cause HAIs, which was consistent with the results of the study by Evelyn et al. [25]. The general consensus was that mobile phones should be cleaned and disinfected regularly. Similarly, a cross-sectional study in Kuwait found that 63.0% of HCWs thought mobile phones could play a role in spreading infections in healthcare settings, but 68.0% of them opposed banning the use of mobile phones in their departments [11]. In the current study, 46.8% of HCWs opposed banning the use of mobile phones at work. A study aimed to investigate the level of phones contamination on surgical wards also showed that 75% of people felt that banning the use of phones would not be a practical or realistic solution to reduce infections [22]. Without HCWs’ support for the banning of mobile phone use in hospitals, it is rather important to improve infection control awareness and mobile phone hygiene practice than simply strict restrictions.

Although the HCWs’ phones were almost completely contaminated, the total number of colonies on the phones was lower than that on HCWs’ hands, which seemed to be different from what is usually thought. Contamination with bacterial pathogens was found in 95.5% of HCWs’ mobile phones, consistent with 94.6 [12] and 96.5% [15] of the bacterial contamination rates reported by other researchers. There was even a study performed in a tertiary-level Italian ICU found that all phones (100%) were positive for bacteria [26]. No molecular tests were conducted to determine the clonal relation in this survey, but the detection rate and the bacterial isolates from HCWs’ hands and mobile phones were highly similar (concordance rate = 38.9%). The cross-contamination between the surfaces of mobile phones and HCWs’ hands might exist. Similar bacterial contamination found on hands and phones is relational because most germs encountered are present in the healthcare environment, including dry surfaces not touched by hands [27]. Perhaps in the future study, we can estimate and predict the hand contamination through mobile phone contamination. A number of researches have demonstrated that the most common Gram-positive bacterium isolated from mobile phone surfaces was *Staphylococcus* and the most common Gram-negative bacterium was *Acinetobacter* [11, 16, 17, 28], consistent with our study. The preponderance of *S. epidermidis* in this study was in accordance with the findings of other researchers [29]. In addition, it has been reported that *Klebsiella pneumoniae* was the predominant pathogen isolated from HCWs’ mobile phones at Felege Hiwot Referral Hospital, northwest Ethiopia [9], but no such result was found in our study. In the hospital setting, although some bacteria which are normal floras on human skins or in the mouths, such as *S. epidermidis*, are considered to be non-pathogenic in normal circumstances, their high levels of presence on mobile phones with frequent hand contact may pose a risk of HAIs. *S. aureus* and *Acinetobacter baumannii* isolated from samples in our study were common causes of HAIs associated with high morbidity and mortality. In a previous study on the microbiome analyses of hospital mobile phones, pathogens including methicillin-resistant *Staphylococcus aureus* (MRSA) and vancomycin-resistant *Enterococcus* (VRE) were isolated [30]. Galazzi A et al. have reported that the isolation rate of MRSA was up to 17% in ICU [26]. Another study from Ethiopia showed that Gram-negative bacteria were isolated from 8.3% of HCWs’ mobile phones and 79.4% of the isolates were multidrug resistant [31]. Antibiotic-resistant bacteria are the most serious health risk for hospitalized patients [32]. Besides, *Enterococcus faecium and E. coli* were isolated from mobile phones in this study, which suggested poor hygiene conditions of mobile phones and hands because these bacteria are part of the intestinal flora.

The non-parametric test showed that there were significant differences of colony count among subgroups of different ages, genders, professions, frequency of mobile phone use, and use of a phone cover. Similarly, for the evaluation of sample cleanliness, the qualified rate of mobile phones of female HCWs was higher than that of males, and the qualified rate of nurses was higher than that of others. It could be explained that most participants were nurses, and they were mainly females, who were better performers of nosocomial infection control measures, such as hand hygiene [33]. A study conducted in East Ethiopia also found that mobile phones of male HCWs were more contaminated [34]. Male HCWs seems to need more external reminders of mobile phone disinfection and hand hygiene than female HCWs. There was an association between mobile phone qualified rate of bacteria and utilization. With the high daily use rate of mobile phones and the less strict hygienic practices of users, it is reasonable to expect that cross-contamination of microorganisms from phones to hands will occur [35]. After adjusted by potential confounders, all the included factors were not statistically associated with phone qualified rate of bacteria except for the frequency of phone utilization. The results may be attributed to the limited sample size. Previous studies have shown that some factors can be related to phone bacterial contamination, including gender, phone usage frequency, type of phone, and medical specialty [13, 36, 37]. But Heyba et al. found that the only factor that was significantly associated with mobile phone bacterial contamination was whether a doctor had disinfected his mobile phone [11]. Japanese scholars have observed that the bacterial contamination rate was negatively correlated with the frequency of hand hygiene [38], so hand hygiene may be a protective factor against mobile phone bacterial contamination. Based on previous researches and our findings, we have reasons to believe that even if mobile phones are contaminated, the risk of HAIs will be significantly reduced in case HCWs take good hand hygiene before and after entering a patient room (or giving medical cares) [39].

For the first time to our knowledge, we found that the numbers of bacterium colonies on mobile phone surfaces with phone covers were significantly larger than those without. People are accustomed to using a phone cover to prevent mobile phone screen from breaking or bumping. If phone covers are not cleaned and disinfected regularly, they will be more conducive to bacterial colonization. We recommend that HCWs should remove mobile phone covers in their facilities, especially in medical activities. The materials of mobile phone covers are mostly silicone, polycarbonate (PC), and thermoplastic urethane (TPU), which are more conducive to microbial growth than glass materials. This may explain why the phone cover grows more bacteria. In future, phone surface coatings may be a promising option to prevent bacterial adhesion and biofilm formation given the prevalence of antibiotic-resistant bacterial strains [40].

## Limitations

There are three limitations in our present study. Firstly, we did not perform antimicrobial susceptibility testing and homology analysis for the isolated organism. Some common bacteria like *Pseudomonas aeruginosa* were not isolated in this study. Secondly, an imbalance among HCWs in favor of nurses occurred within sampling procedure. It may impact the generalizability of our findings to other HCWs. Thirdly, some behavioral factors that may affect the transmission of pathogens were not considered, such as hand-to-mouth and other hand-to-face touching behaviors which may further the transfer of pathogenic microbial agents.

## Conclusion

In conclusion, our study provided more evidence that HCWs’ mobile phones were highly contaminated with various bacteria in hospitals, and the bacterial contamination between mobile phones and hands was closely related. Most of the isolates were common pathogens or conditional pathogens related to HAIs. Age, gender, profession, phone cover and the frequency of mobile phone utilization were the significantly associated factors of bacterial contamination of mobile phones in this study. More contact with phone screens would increase the bacterial contamination of mobile phones.

Based on the above findings, we strongly recommend standardizing the use of mobile phones in key departments, formulating relevant cleaning guidelines, and increasing the awareness about mobile phone disinfection in healthcare settings. HCWs should remove mobile phone covers in medical activities, if possible. Furthermore, the disinfection of the mobile phone cover and strict hand hygiene are also essential.

## Supplementary Information

Below is the link to the electronic supplementary material.

Supplementary file1 Table S1 Comparison of qualified rate and bacterial load of mobile phones according to other variables (DOCX 22 KB)

## Data Availability

All raw data are available upon reasonable request from the primary author.

## Abbreviations

HAIs: Hospital-acquired infections
HCWs: Healthcare workers
*CoNS*: *Coagulase-negative staphylococci*
ICU: Intensive care unit
CFU: Colony-forming unit
IQR: Interquartile range
*S.*: *Staphylococcus*
*E.coli*: *Escherichia coli*
MRSA: Methicillin-resistant *Staphylococcus aureus*
VRE: Vancomycin-resistant *Enterococcus*
