# Supplementary material for: Bacterial Colonization on Healthcare Workers’ Mobile Phones and Hands in Municipal Hospitals of Chongqing, China: Cross-contamination and Associated Factors
Source: J Epidemiol Glob Health. 2022 Sep 7;12(4):390–9. doi: 10.1007/s44197-022-00057-1 (PMC9449931; doi:10.1007/s44197-022-00057-1)
Supplement: Supplementary file 1 — Supplementary file1 Table S1 Comparison of qualified rate and bacterial load of mobile phones according to other variables (DOCX 22 KB) [file 44197_2022_57_MOESM1_ESM.docx]

**Table S1** Comparison of qualified rate and bacterial load of mobile phones according to other variables.

| **Characteristics and usage** | | **No. of HCWs**  **(N=111)** | **No. of qualified** | **Qualified rate (%)** | ***χ^2^*** | ***p*** | **Median No. of**  **Colonies (IQR) (CFU/cm^2^)** | ***Z*** | ***p*** |
| --- | --- | --- | --- | --- | --- | --- | --- | --- | --- |
| A smart phone or not | Yes | 110 | 88 | 80.0 | - | 1.000^a^ | 2.9 (0.7—6.7) | -1.06 | 0.291 |
|  | No | 1 | 1 | 100.0 |  |  | 0.6 (0.6—0.6) |  |  |
| Size of the phone screen | ＜ 5.0 inches | 68 | 56 | 82.4 | 0.52 | 0.470 | 2.2 (0.7—5.6) | 0.85 | 0.397 |
|  | ≥ 5.0 inches | 43 | 33 | 76.7 |  |  | 2.9 (0.8—7.2) |  |  |
| Daily mobile phone usage time | ＜1 hour | 12 | 8 | 66.7 | 1.57 | 0.456 | 4.2 (0.9—11.6) | 1.15^b^ | 0.563 |
|  | 1—2 hours | 26 | 21 | 80.8 |  |  | 3.1 (1.1—5.6) |  |  |
|  | ＞ 2 hours | 73 | 60 | 82.2 |  |  | 1.9 (0.6—6.7) |  |  |
| Use of mobile phone at eating | Yes | 40 | 36 | 90.0 | 3.79 | 0.051 | 1.2 (0.6—4.8) | -1.76 | 0.081 |
|  | No | 71 | 53 | 74.7 |  |  | 2.9 (1.1—8.4) |  |  |
| Use of mobile phone before sleep | Yes | 97 | 80 | 82.5 | 2.55 | 0.111 | 2.5 (0.7—5.9) | 1.30 | 0.196 |
|  | No | 14 | 9 | 64.3 |  |  | 4.5 (1.8—8.8) |  |  |
| Use of mobile phone in the bathroom | Yes | 51 | 43 | 84.3 | 1.01 | 0.314 | 2.1 (0.6—5.2) | -0.81 | 0.418 |
|  | No | 60 | 46 | 76.7 |  |  | 3.1 (0.9—7.5) |  |  |
| Use of mobile phone at work | Yes | 27 | 24 | 88.9 | 1.70 | 0.192 | 1.5 (0.6—4.4) | -1.23 | 0.222 |
|  | No | 84 | 65 | 77.4 |  |  | 3.3 (0.7—7.5) |  |  |
| Mobile phone use is banned in the department | Yes | 71 | 57 | 80.3 | 0.001 | 0.972 | 2.9 (0.7—7.9) | -0.42 | 0.675 |
|  | No | 40 | 32 | 80.0 |  |  | 2.6 (0.6—4.8) |  |  |
| Frequency of mobile phone cleaning and disinfection | Regularly | 36 | 31 | 86.1 | 3.16 | 0.206 | 1.7 (0.5—4.7) | 3.85^b^ | 0.146 |
|  | Occasionally | 68 | 54 | 79.4 |  |  | 3.1 (0.7—7.7) |  |  |
|  | Never | 7 | 4 | 57.1 |  |  | 3.4 (2.9—48.2) |  |  |
| The way used in mobile phone disinfection^c^ | Wipe with alcohol | 78 | 62 | 79.5 | 6.15 | 0.105^d^ | 2.4 (0.8—6.8) | 7.61^b^ | 0.055 |
|  | Wipe with wet tissue | 21 | 20 | 95.2 |  |  | 1.7 (0.3—3.8) |  |  |
|  | Wipe with a napkin | 4 | 2 | 50.0 |  |  | 8.2 (3.7—27.0) |  |  |
|  | Else | 1 | 1 | 100.0 |  |  | 0.05 (0.05—0.05) |  |  |
| Hands cleaning while cleaning mobile phone^c^ | Yes | 81 | 68 | 84.0 | 1.21 | 0.272 | 2.3 (0.7—5.6) | 1.04 | 0.300 |
|  | No | 23 | 17 | 73.9 |  |  | 2.5 (0.8—10.3) |  |  |
| Do you think your phone is contaminated with bacteria? | Yes | 109 | 87 | 79.8 | - | 1.000^a^ | 2.9 (0.7—6.7) | -0.30 | 0.765 |
|  | No | 2 | 2 | 100.0 |  |  | 2.1 (0.7—3.5) |  |  |
| Do you think your phone should be cleaned and disinfected regularly? | Yes | 106 | 86 | 81.1 | - | 0.257^a^ | 2.9 (0.7—6.6) | 0.63 | 0.528 |
|  | No | 5 | 3 | 60.0 |  |  | 2.1 (1.1—30.9) |  |  |
| Do you think the phone should be banned at work in your department? | Yes | 59 | 49 | 83.1 | 0.65 | 0.419 | 1.9 (0.6—5.9) | 1.31 | 0.192 |
|  | No | 52 | 40 | 76.9 |  |  | 3.2 (0.8—8.4) |  |  |
| Do you think the phone could cause HAI? | Yes | 98 | 80 | 81.6 | 1.11 | 0.292 | 2.7 (0.6—6.6) | 1.53 | 0.130 |
|  | No | 13 | 9 | 69.2 |  |  | 3.5 (1.3—17.0) |  |  |
| Do you think the phone could spread diseases? | Yes | 96 | 77 | 80.2 | - | 1.000^a^ | 2.9 (0.7—6.8) | 0.41 | 0.683 |
|  | No | 15 | 12 | 80.00 |  |  | 2.3 (0.9—6.2) |  |  |

IQR interquartile range; CFU colony-forming unit

^a^ Fisher’s exact test

^b^ The *χ^2^* of Kruskal-Wallis test

^c^ The total number of HCWs answered for this question was 104 (There were 7 people who had never cleaned or disinfected their phones)

^d^ Continuous-Corrected Chi-Squared test
